# Drug susceptibility patterns of *Mycobacterium tuberculosis* from adults with multidrug-resistant tuberculosis and implications for a household contact preventive therapy trial

**DOI:** 10.1186/s12879-021-05884-4

**Published:** 2021-02-24

**Authors:** Anne-Marie Demers, Soyeon Kim, Sara McCallum, Kathleen Eisenach, Michael Hughes, Linda Naini, Alberto Mendoza-Ticona, Neeta Pradhan, Kim Narunsky, Selvamuthu Poongulali, Sharlaa Badal-Faesen, Caryn Upton, Elizabeth Smith, N. Sarita Shah, Gavin Churchyard, Amita Gupta, Anneke Hesseling, Susan Swindells, for the ACTG A5300/IMPAACT I2003 PHOENIx Feasibility study team

**Affiliations:** 1 Desmond Tutu TB Centre, Department of Paediatrics and Child Health, Faculty of Medicine and Health Sciences, Stellenbosch University, Cape Town, South Africa; 2 Department of Biostatistics, Frontier Science Foundation, Brookline, MA USA; 3 Harvard T.H. Chan School of Public Health, Boston, MA USA; 4 TB or NOT TB Consulting, LLC, Little Rock, AR USA; 5 Social & Scientific Systems, Inc., Silver Spring, MD USA; 6 Barranco Clinical Research Site, Lima, Peru; 7 Byramjee Jeejeebhoy Government Medical College-Johns Hopkins University Clinical Research Site, Pune, Maharashtra India; 8 UCTLI, Cape Town, South Africa; 9 Chennai Antiviral Research and Treatment (CART) Clinical Research Site, Infectious Diseases Medical Center, Voluntary Health Services, Chennai, India; 10 University of the Witwatersrand Helen Joseph (WITS HJH) CRS, Johannesburg, South Africa; 11 TASK Applied Science, Cape Town, South Africa; 12 DAIDS, NIH, Bethesda, MD USA; 13 Centers for Disease Control and Prevention, Atlanta, GA USA; 14 Aurum Institute, Parktown, South Africa; 15 School of Public Health, University of Witwatersrand, Johannesburg, South Africa; 16 Department of Medicine, Johns Hopkins University, Baltimore, MD USA; 17 Department of Internal Medicine, Section of Infectious Diseases, University of Nebraska Medical Center, Omaha, NE 68198-8106 USA

**Keywords:** Tuberculosis, Drug-resistance, Antimicrobial susceptibility testing, Trial, Preventive therapy

## Abstract

**Background:**

Drug susceptibility testing (DST) patterns of *Mycobacterium tuberculosis* (MTB) from patients with rifampicin-resistant tuberculosis (RR-TB) or multidrug-resistant TB (MDR-TB; or resistant to rifampicin and isoniazid (INH)), are important to guide preventive therapy for their household contacts (HHCs).

**Methods:**

As part of a feasibility study done in preparation for an MDR-TB preventive therapy trial in HHCs, smear, Xpert MTB/RIF, Hain MTBDR*plus*, culture and DST results of index MDR-TB patients were obtained from routine TB programs. A sputum sample was collected at study entry and evaluated by the same tests. Not all tests were performed on all specimens due to variations in test availability.

**Results:**

Three hundred eight adults with reported RR/MDR-TB were enrolled from 16 participating sites in 8 countries. Their median age was 36 years, and 36% were HIV-infected. Routine testing on all 308 were confirmed as having RR-TB, but only 75% were documented as having MDR-TB. The majority of those not classified as having MDR-TB were because only rifampicin resistance was tested. At study entry (median 59 days after MDR-TB treatment initiation), 280 participants (91%) were able to produce sputum for the study, of whom 147 (53%) still had detectable MTB. All but 2 of these 147 had rifampicin DST done, with resistance detected in 89%. Almost half (47%) of the 147 specimens had INH DST done, with 83% resistance. Therefore, 20% of the 280 study specimens had MDR-TB confirmed. Overall, DST for second-line drugs were available in only 35% of the 308 routine specimens and 15% of 280 study specimens.

**Conclusions:**

RR-TB was detected in all routine specimens but only 75% had documented MDR-TB, illustrating the need for expanded DST beyond Xpert MTB/RIF to target preventive therapy for HHC.

## Background

In 2018, globally an estimated 484,000 people developed TB with strains resistant to rifampicin (RIF), and of these, 78% had resistance to both RIF and isoniazid (INH), or multidrug-resistant TB (MDR-TB) [1]. In recent years, rapid and sensitive tests based on molecular methods, including Xpert MTB/RIF (Xpert, Cepheid, Sunnyvale USA) and line probe assays (LPA) such as the Hain GenoType MTBDR*plus* assay (Hain, Hain Lifescience, Nehren, Germany), have been endorsed by the World Health Organization (WHO) and have become routinely available in TB programs in several countries. Diagnostic algorithms for MDR-TB vary across countries [2].

The optimal preventive therapy regimen for people exposed to MDR-TB is not known and evidence based guidelines are urgently needed. Current WHO guidelines recommend that the preventive treatment should be individualized after a careful assessment of the intensity of exposure, the certainty of the source case, reliable information on the drug resistance pattern of the source case and potential adverse events. They also acknowledge the lack of quality evidence and specifically recommend clinical trials as a high priority. Three large phase 3 trials are underway to address this question. Two are evaluating levofloxacin versus placebo: TB-CHAMP (ISRCTN92634082) and VQUIN MDR (ACTRN12616000215426), and the PHOENIx trial comparing the efficacy and safety of delamanid versus isoniazid (NCT03568383). We conducted a feasibility study in preparation for the PHOENIx trial The feasibility study evaluated index cases with reported MDR-TB and their HHCs at 16 sites, in eight high TB burden countries [3]. The objectives of the feasibility study were to (1) identify, recruit, and characterize adult MDR-TB index cases and their adult and child HHCs; (2) describe the prevalence of TB disease, TBI, and HIV among HHC; and (3) estimate the proportion of HHCs at high risk of TB and, therefore, potentially eligible for the interventional trial. Briefly, we found that participating sites were readily able to find and recruit patients with MDR-TB and their HHC. Many of the latter had either prevalent TB infection or disease, or were otherwise at high risk for TB, and very few were receiving preventive therapy. The mycobacteriology objectives for this publication were to determine if MTB was detectable and if resistance to INH and RIF could be confirmed, genotypically and phenotypically, in the adult index cases with drug resistant TB at time of enrolment. Here we report the mycobacteriology results and discuss how they informed the trial design, as well as their public health implications.

## Methods

### Study participants

In this cross-sectional study, index cases were adults 18 years or older with pulmonary MDR-TB or rifampicin-resistant-TB (RR-TB) by phenotypic or genotypic testing. Index cases identified by the site or local TB program with pulmonary MDR/RR-TB were all approached for enrollment if they met the following additional inclusion criteria: (1) initiated MDR-TB treatment within 6 months prior to study enrollment, (3) had at least one household contact (HHC), (4) provided permission to enumerate and screen HHCs, and (5) resided at a distance deemed by the site study team close enough for study conduct. The study enrolment period was October 2015 to April 2016. TB treatment was provided by the routine TB programs according to local guidelines. The study was approved by site Institutional Review Boards or Ethics Committees and all participants gave written informed consent.

### Study procedures

The mycobacteriology results from sputum specimens from the routine program (hereafter referred to as routine specimens) for MDR/RR-TB diagnosis were recorded; these included smear microscopy, Xpert, LPAs, culture and DST. Not all tests were performed across sites due to variations in test availability. A sputum sample was collected at study entry (hereafter referred to as study specimens). Smear microscopy (GLI-WHO-IUATLD grading scale [4]), Xpert, and liquid culture using MGIT (Mycobacteria Growth Indicator Tube, BD Diagnostics, Sparks, MD) were performed. Solid culture was optional. Positive cultures were identified using an MPT64 antigen assay and/or Hain MTBDR*plus,* as the latter was not available in all participating laboratories. When done, phenotypic DST for first and second line TB drugs was performed by either MGIT or another indirect proportion method using WHO critical concentrations [5] at some sites.

### Definitions

Time since RR/MDR-TB diagnosis for microbiology testing was defined as the interval between the date of treatment initiation and the date the study specimen was obtained. Pre-extensively drug-resistant TB (Pre-XDR-TB) was defined as MDR-TB with resistance to fluoroquinolones (FLQ) or second-line injectable drugs (SLID). XDR-TB was defined as MDR-TB plus resistance to any fluoroquinolones and any of the SLID [1]. When multiple specimens were available, results were classified based on the “worst” result; for example, if one smear was positive and the others negative, the overall smear result was classified as positive. For culture, determinate results were prioritized over those contaminated. For both routine and study specimens, if DST results were obtained by several methods, the overall result was classified as resistant if reported as such by at least one method. Results were described as discordant when DST results for one drug were different when tested by various methods (between molecular assays and/or phenotypically).

### Statistical considerations

All summaries are descriptive. We calculated simple proportions for categorical variables and medians (interquartile ranges (IQR)) for quantitative variables.

## Results

### Study participants

Three hundred twenty-eight potential participants were approached of whom 321 agreed to be screened. Three hundred eight were enrolled between October 2015 and March 2016 from 16 participating sites in 8 countries: Botswana, Brazil, Haiti, India, Kenya, Peru, South Africa, and Thailand. All sites are clinical research sites of the AIDS Clinical Trials Group (ACTG) and/or the International Maternal Pediatric Adolescent AIDS Clinical Trials (IMPAACT) networks. The median age was 36 years and 57% were male; 41% of participants were black, 32% mixed race/other, 22% Asian, and 2% white; 112 (36%) were HIV-infected; and 43% were current or former smokers. 87 (43%) had documented cavitary pulmonary disease and 161 (52%) had received prior TB treatment.

### Quantity and timing of specimen collection

The 308 participants had one to 4 sputum sample results from the routine program available which had established the MDR-TB diagnosis, for a total of 404 specimens recorded. Of the 308 participants, 27 (9%) could not produce sputum, 1 declined to provide a study sample, and the remaining 280 (91%) had a study sputum specimen collected at a median (range) of 59 days (0, 190) after MDR-TB treatment initiation.

### Smear and MTB detection by Xpert, Hain and culture

For routine specimens, 217/308 (71%) of participants had smear results available: 69% (149/217) were positive. All study specimens underwent smear testing; only 34% (94/280) were acid-fast bacilli positive, with lower smear grading values (Table 1). Xpert results of routine specimens were available for 152/308 (49%) participants and all but 2 reported MTB. Almost all study specimens were tested by Xpert (99%; 278/280) but detected MTB in only 51% (141/278). Hain testing of routine specimens or positive cultures was reported in 69% (214/308), with MTB detected in 99%. Hain testing was performed on only 27 study specimens or positive cultures (10%; 27/280) and detected MTB in 21 (78%; 21/27). Liquid and/or solid culture results were available for 63% (193/308) routine and 98% (274/280) study specimens while reported positive for MTB in 93% (179/193) and 31% (85/275), respectively. Overall, MTB was detected by Xpert, Hain and/or culture in all participants from routine specimens, and in 53% (147/280) of study specimens collected on treatment.

**Table 1:** Smear, Xpert MTB/RIF, Hain MTBDR*plus* and culture results for MDR-TB index cases from routine and study sputum specimens

	Routine sputum used by TB program for MDR-TB diagnosis (combined specimens per index case)			Study sputum collected on MDR-TB treatment (one specimen per index case)		
	n	% of done	% of 308	n	% of done	% of 280
Smear done^a^	217	–	70.5	280	–	100.0
Smear positive	149	68.7	48.4	94	33.6	33.6
Smear 3+	57	26.3	18.5	20	7.1	7.1
Smear 2+	39	18.0	12.7	18	6.4	6.4
Smear 1+	41	18.9	13.3	19	6.8	6.8
Smear scanty	12	5.5	3.9	37	13.2	13.2
Smear negative	68	31.3	22.1	186	66.4	66.4
Xpert done	152	–	49.4	278	–	99.3
Xpert MTB+	150	98.7	48.7	141	50.7	50.4
Xpert MTB not detected	1	0.7	0.3	135	48.6	48.2
Xpert no result	1	0.7	0.3	2	0.7	0.7
Hain MTBDR*plus* done	214	–	69.5	27	–	9.6
Hain MTBDR*plus* MTB+	211	98.6	68.5	21	77.8	7.5
Hain MTBDR*plus* MTB not detected	2	0.9	0.6	6	22.2	2.1
Hain MTBDR*plus* indeterminate	1	0.5	0.3			
Liquid culture done	159	–	51.6	274	–	97.9
Liquid culture MTB+	147	92.5	47.7	82	29.9	29.3
Liquid culture NTM+				3	1.1	1.1
Liquid culture negative	8	5.0	2.6	168	61.3	60.0
Liquid culture contaminated	4	2.5	1.3	21	7.7	7.5
Solid culture done	64	–	20.8	58	–	20.7
Solid culture MTB+	57	89.1	18.5	14	24.1	5.0
Solid culture negative	5	7.8	1.6	43	74.1	15.4
Solid culture contaminated	2	3.1	0.6	1	1.7	0.4
Liquid or solid culture done	193	–	62.7	275	–	98.2
Liquid or solid culture MTB+	179	92.7	58.1	85	30.9	30.4
Liquid or solid culture negative	9	4.7	2.9	172	62.5	61.4
Liquid or solid culture contaminated	5	2.6	1.6	18	6.5	6.4
Any Xpert or Hain or culture done	308	–	100.0	280	–	100.0
Any Xpert or Hain or culture MTB+	308	100.0	100.0	147	52.5	52.5

*MTB+ M.tuberculosis* complex detected or positive, *NTM* nontuberculous mycobacteria

^a^Smear microscopy using GLI-WHO-IUATLD grading scale [4], smear positive includes scanty, 1+, 2+, and 3+

### Rifampicin susceptibility testing

**Fig. 1:**
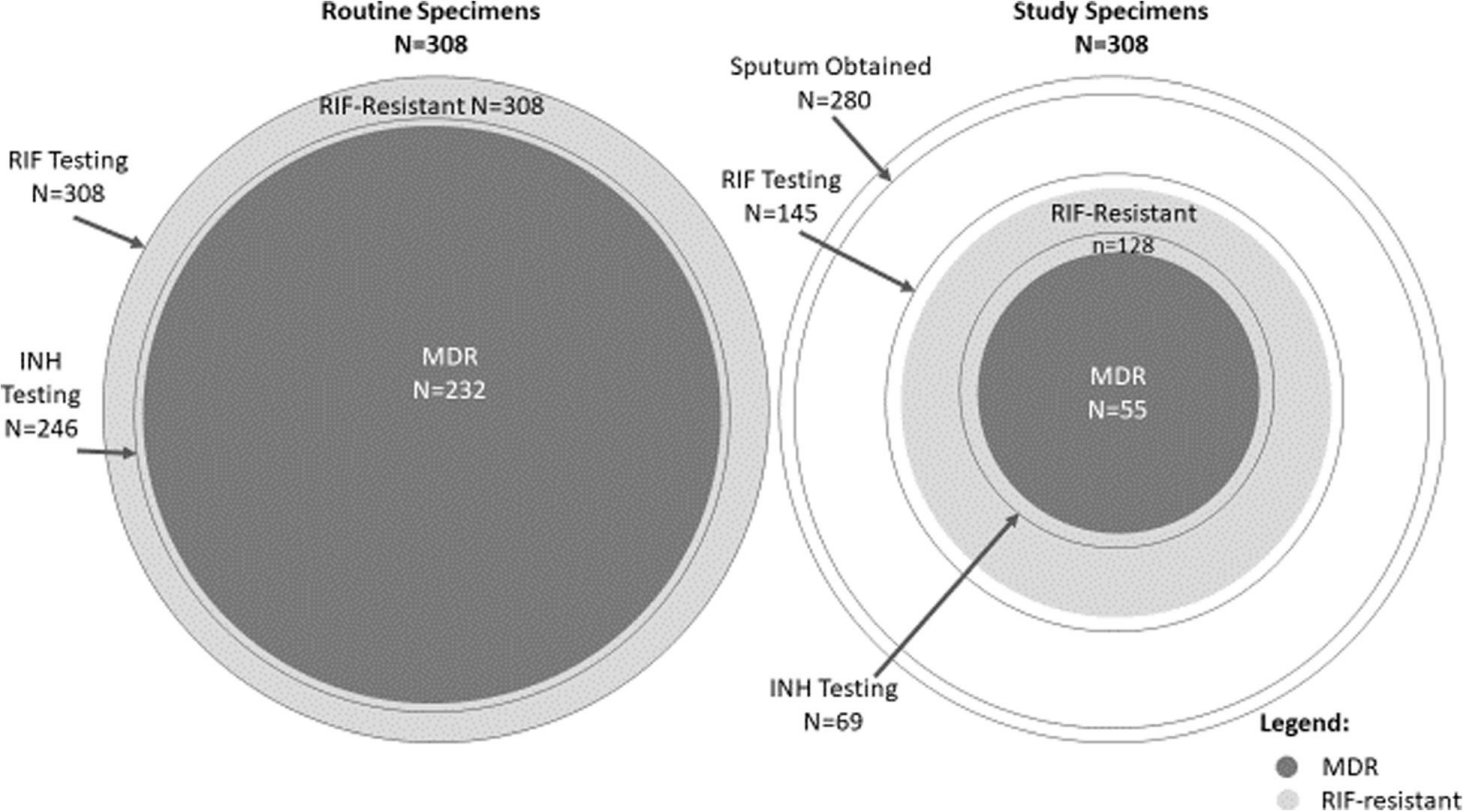
Venn diagram of routine and study specimens showing proportion with documented MDR-TB, and details of INH and RIF drug susceptibility testing. Legend: Drug susceptibility testing was performed by molecular and/or phenotypic methods. Of 280 participants providing on-treatment sputum specimens, MTB was not identified in 133 and so DST could not be performed, MTB was identified but no RIF DST was performed in 2, and MTB was identified and RIF DST was performed in 145 participants. The area of circles are proportional to the frequencies

RIF DST results for routine and study specimens are shown by testing method in Table 2, and combining Xpert, Hain and phenotypic results in Table 3 and Fig. 1**.** In the routine specimens positive for MTB, RIF resistance was detected in 99, 99 and 100% when tested by Xpert, Hain and phenotypic DST, respectively. If RIF resistance was not detected by one method, it was demonstrated by another. Overall for all combined methods, there was evidence of RIF resistance in all 308 routine specimens. For study specimens with MTB detected, RIF resistance was detected in 89, 81 and 89% of those tested by Xpert, Hain and phenotypic DST, respectively. Susceptible and/or discordant results were observed for all 3 methods. Fourteen RIF susceptible Xpert results were recorded: 12 were not tested by any other method while 2 were also susceptible phenotypically. Three study specimens had RIF resistance detected by Xpert but were RIF susceptible phenotypically (Table 3). Overall, of the 280 study specimens, 147 (53%) had MTB detected, and of these, 145 had RIF susceptibility testing done by either methods, of which 128 (89%) had evidence of RIF resistance. Therefore, 46% of 280 study specimens had RIF resistance documented.

**Table 2:** Rifampicin (RIF), Isoniazid (INH) and second-line drug susceptibility testing methods and results for MDR-TB index cases from routine and study sputum specimens

	MDR-TB Diagnosis by routine program (combined specimens per index case)			Study sputum collected after start of MDR-TB treatment (one specimen per index case)		
	n	% of done	% of 308 IC	n	% of done	% of 280 IC
MTB detection (from Table 1)						
Xpert MTB+	150	98.7	48.7	141	50.7	50.4
Hain MTBDR*plus* MTB+	211	98.6	68.5	21	77.8	7.5
Liquid or solid culture MTB+	179	92.7	58.1	85	30.9	30.4
Any Xpert or Hain or culture MTB+	308	100.0	100.0	147	52.5	52.5
RIF susceptibility testing						
Done by Xpert Hain and/or pheno	308		100.0	145		51.8
Resistant by Xpert and/or Hain and/or pheno	308	100.0	100.0	128	88.3	45.7
Done by Xpert	150		48.7	141		50.4
Resistant by Xpert	148	98.7	48.1	125	88.7	44.6
Done by Hain	211		68.5	21		7.5
Resistant by Hain	209	99.1	67.9	17	81.0	6.1
Done by pheno	102		33.1	53		18.9
Resistant by pheno	102	100.0	33.1	47	88.7	16.8
Done by pheno only	29		9.4	3		1.1
INH susceptibility testing						
Done by Hain and/or pheno	246		79.9	69		24.6
Resistant by Hain and/or pheno	232	94.3	75.3	57	82.6	20.4
Done by Hain	204		66.2	21		7.5
Resistant by Hain	185	90.7	60.1	14	66.7	5.0
Done by pheno	101		32.8	54		19.3
Resistant by pheno	100	99.0	32.5	49	90.7	17.5
Done by pheno only	42		13.6	48		17.1
FLQ susceptibility testing						
Done by Hain and/or pheno	108		35.1	41		14.6
Resistant by Hain and/or pheno	7	6.5	2.3	6		14.6
Streptomycin susceptibility testing						
Done by pheno	29		9.4	54		19.3
Resistant by pheno	11	37.9	3.6	39	72.2	13.9
SLID susceptibility testing						
Done by Hain and/or pheno	107		34.7	42		15
Resistant by Hain and/or pheno	8	7.5	2.6	5	11.9	1.8

*FLQ* fluoroquinolones (FLQ), *MTB+ M.tuberculosis* complex detected or positive, *pheno* phenotypic drug susceptibility testing, *SLID* second-line injectable drugs

**Table 3:** MDR status of Index Cases from routine and study sputum specimens. Detailed results for rifampicin (RIF) and isoniazid (INH) are provided on the left with combined MDR status on the right

Rifampicin Resistance	Isoniazid Resistance	Routine n	Study n	MDR status^**a**^	Routine		Study	
					n	% of 308	n	% of 280
R: Hain	R: Hain	86		**MDR**	**232**	**75.3**	**55**	**19.6**
R: Hain	Discord: Hain S, Pheno R	1						
R: Hain & Pheno	R: Hain & Pheno	32						
R: Hain & Pheno	Discord: Hain S, Pheno R	1						
R: Pheno	R: Pheno	28	3					
R: Xpert	R: Pheno		1					
R: Xpert & Hain	R: Hain	46	8					
R: Xpert & Hain	Discord: Hain S, Pheno R	2						
R: Xpert & Pheno	R: Pheno	12	35					
R: Xpert, Hain, & Pheno	R: Hain & Pheno	21	6					
R: Xpert, Hain, & Pheno	Discord: Hain S, Pheno R	1						
Discord: Hain S, Pheno R	Discord: Hain S, Pheno R	1						
Discord: Xpert R, Pheno R and S^b^	Discord: Pheno R and S^b^		1					
Discord: Xpert R, Pheno S^c^	R: Pheno		1					
Discord: Xpert S, Pheno R	R: Pheno	1						
R: Hain	I: Hain	1		**RIF R, INH not R**	**13**	**4.2**	**6**	**2.1**
R: Xpert & Hain	I: Hain		1					
Discord: Xpert R, Hain I	I: Hain		1					
Discord: Xpert R, Hain S	I: Hain		1					
R: Xpert & Hain	S: Hain	11	2					
Discord: Xpert R, Hain S	S: Hain	1	1					
R: Hain	(no results)	7		**RIF R, INH no results**	**62**	**20.1**	**63**	**22.5**
R: Xpert	(no results)	51	63					
R: Xpert & Pheno	(no results)	3						
Discord: Xpert S, Pheno R	(no results)	1						
R: Pheno	S: Pheno	1		**Mono-R to RIF(S to INH)**	**1**	**0.3**	**4**	**1.4**
R: Xpert & Pheno	S: Pheno		2					
Discord: Xpert R, Pheno S^c^	S: Pheno		2					
S: Xpert & Pheno	R: Pheno		2	**Mono-R to INH(S to RIF)**			**2**	**0.7**
S: Hain	S: Hain		1	**RIF not R, INH not R**			**2**	**0.7**
Discord: Xpert I, Pheno S	S: Pheno		1					
I: Xpert	(no results)		1	**RIF not R, INH no results**			**13**	**4.6**
S: Xpert	(no results)		12					
(no results)	(no results)		135	**No results**			**135**	**48.2**
**TOTAL**		**308**	**280**		**308**	**100.0**	**280**	**100.0**

*R* Resistant; S = Susceptible, *I* Indeterminate, *Discord* Discordant results

*Hain* resistance detected by Hain, *Pheno* resistance detected phenotypically, *Xpert* resistance detected by Xpert

^a^Classified as resistant (R) if resistance reported by any method. If not resistant, noted as “not R”

^b^Pheno R and S: Indirect proportion R, MGIT S

^c^Xpert R, Pheno S by MGIT: could be disputed rpoB mutations

### INH susceptibility testing

For routine specimens with MTB detected, INH resistance was detected in 91, and 99% of those tested by Hain and phenotypic DST, respectively (Table 2). Susceptible and discordant results were observed: six participants were INH susceptible by Hain testing but resistant phenotypically (Table 3). Overall, all 308 routine specimens had MTB detected. Of these, 246 (80%) had INH susceptibility testing done by either method, of which 232 (94%) had evidence of INH resistance. Therefore, 75% of routine specimens had INH resistance documented (Table 2). For the study specimens with MTB detected, INH resistance was detected in 67 and 91% tested by Hain and phenotypic DST, respectively. Susceptible and/or discordant results were observed such as 4 INH susceptible by Hain which were however not tested by phenotypic DST. There were 5 INH susceptible results phenotypically tested and not by Hain (Table 3), Overall, for the 280 study specimens, 147 had MTB detected and of these, 69 (25% of 280) had INH DST done by either method, of which 57 (83% of 69) had evidence of INH resistance. Therefore, only 20% of study specimens had INH resistance documented (Table 2).

### Fluoroquinolones (FLQ) and injectable drug susceptibility testing

Only 108 (35%) of the 308 participants had FLQ susceptibility results available on routine specimens by either Hain or phenotypic DST since this was not standard in most countries: 7 (6%) demonstrated resistance. Of 280 study specimens, only 41 (15%) had FLQ susceptibility results, with 6/41 (15%) demonstrating resistance (data not shown). Streptomycin resistance was observed in 38 and 72% of the 29 and 54 routine and study specimens, respectively. For SLIDs, only 107 (35%) of routine specimens had susceptibility testing done and results by either Hain or phenotypic DST, with resistance detected in 8 (7%). For study specimens, only 42 (15%) had SLID susceptibility results, with 5 (12%) resistant.

### MDR and XDR status

Detailed results for INH and RIF for routine and study specimens are shown in Table 3 and Fig. 1. MDR-TB was confirmed in 75% (232) of 308 routine specimens. In study specimens, MTB was detected by Xpert, Hain or culture in 50, 8 and 30%, respectively (Table 2 and Table 3) and with INH susceptibility testing being only possible by Hain or phenotypic DST, only 20% (55/280) had MDR-TB confirmed. Monoresistance to INH (0% in routine, 0.7% in study samples) and monoresistance to RIF (0.3% in routine and 1.4% in study samples) by phenotypic testing was rarely documented. In 20% of routine and 23% of study specimens, only RIF resistance was documented (mainly by Xpert) with no susceptibility results documented for INH. When adding FLQ and SLID information to the MDR status, 43% (131/308) of the routine specimens and 8.2% (23/280) of the study specimens had MDR confirmed but no results for FLQ and SLID (data not shown). Only 35% of routine specimens and 14% study specimens had testing sufficient to determine participants’ pre-XDR and XDR status. Approximately a third (88/308; 29%) of routine specimens and 9% (24/280) of study specimens had MDR with documented susceptibility to FLQ and SLID. Very few had pre-XDR (7/108 (7%) routine and 5/39 (13%) study) or XDR-TB (4/108 (4%) of routine and 2/39 (5%) of study) (data not shown).

## Discussion

RR-TB was detected in all routinely collected specimens, but only 75% had documented MDR-TB, illustrating the need for expanded DST beyond Xpert MTB/RIF in order to target preventive therapy for HHCs. In many countries, this may require significant capacity building. Only about one-third of participants had sputum specimens collected for the study that grew MTB in culture, thus confirmation of MDR status or expanded DST post-treatment initiation may not be possible in the majority of cases. Moreover, this may hinder future comparisons of genotype and DST patterns between index cases and their household contacts for potential scientific investigations.

In our study, we observed considerable heterogeneity in the testing done on routine and study specimens for MDR-TB patients across 8 countries, with various combinations of molecular and/or phenotypic drug susceptibility tests done in routine care. However, these findings were useful and informed the design and implementation of the interventional trial in several aspects.

The first finding illustrates the challenges related to completing INH DST to confirm MDR status. Routine testing on all 308 index cases confirmed MTB that was resistant to rifampicin, but only three-quarters had evidence of MDR-TB. The majority of those not classified as MDR-TB were because only RIF resistance was tested, mainly by Xpert (Table 3), i.e. RR-TB. According to WHO guidelines, patients with RR-TB should receive MDR-TB treatment regimens, and MDR-TB and RR-TB recommendations are typically grouped together [6]. Whether or not INH DST is needed to determine the ideal treatment regimen for disease has been debated previously [7]. The WHO now recommends that all countries move towards universal testing for both isoniazid and rifampicin resistance at the start of TB treatment [6]. From the perspective of selecting appropriate preventive treatment for close contacts, the drug resistance pattern of the source case is however an important factor [8], since HHCs with exposure to MTB susceptible to INH would benefit from INH-containing regimens Despite receiving MDR-TB treatment for a median of 59 days, 91% of participants were still able to produce sputum at enrollment into the feasibility study, of whom only 53% still had detectable MTB and 20% had MDR-TB confirmed. Of note, Hain testing was optional for the study as it was not available in all network laboratories. After learning that a significant proportion of patients on treatment for MDR-TB only had evidence of RR-TB, we therefore decided to make the documentation of resistance to both RIF and INH in the interventional trial essential, as the treatment arms include delamanid and INH.

The second finding concerns the challenges of completing second line DST. DST results for fluoroquinolones and SLID were limited, so conclusions on pre-XDR and XDR status should be interpreted with caution. Only 35% of participants had DST performed for second-line drugs by the routine TB program. This proportion is likely to become higher as WHO reports that FLQ and SLID DST in MDR/RR-TB patients is becoming more available, increasing from 49% in 2017 to 59% in 2018 [1]. Nine percent of participants could not produce sputum on study. For the 280 participants providing study specimens, MTB was detected by Hain or culture in 8 and 30%, leaving only 14% of study specimens with testing sufficient to determine pre-XDR and/or XDR status. This highlights the additional challenge of performing further DST on specimens collected after MDR-TB treatment initiation, where the lower bacillary load decreases diagnostic yield. All these limitations have important public health consequences for the appropriate management of XDR-TB.

The third finding regarding the interpretation of discordant results is complex, especially when testing is done in specimens obtained at different times and tested in different laboratories [9]. For example, all routine specimens were RIF resistant but 17 study specimens had susceptible or indeterminate RIF results (Table 3). These could possibly represent mutations missed by the assay (which may be improved by use of the updated Ultra version [10]), mixed infections with multiple similar strains, or microevolution of strains within the host [11] under treatment pressure. There were also three Xpert RIF resistant but MGIT susceptible results in study specimens (Table 3); these could in fact be false susceptible MGIT RIF results due to disputed mutations in the *rpoB* gene [12]. Discordant results were also observed for INH: six routine specimens were INH susceptible by Hain but INH resistant phenotypically; this is a known limitation of the Hain MTBDR*plus* assay, which detects only resistance mediated by *katG* or *inhA* mutations [13] or 85% of isoniazid resistance detected by MGIT [14]. Consequently for the intervention trial, a pragmatic approach using any resistance for RIF and for INH was adopted for the Index Case MDR-TB eligibility criteria, as long as considered resistant by the program at the time of evaluation.

The fourth finding was related to the fact that only one-third of participants had culture positive sputum when approached for the study. This would likely impact the planned interventional study objective of comparing genotype and DST patterns between index cases and their household contacts. We shortened the time since MDR diagnosis from 6 months to 3 months for the index case in the interventional trial, which should increase the rate of culture positivity at study enrollment, implying that HHCs would have had significant ongoing MTB exposure at the time of enrolment.

A fifth finding was that among those tested, the rate of smear positivity was high at 69% in routine specimens. A third (34%) of study specimens were still smear positive a median of 59 days after MDR-TB treatment initiation, highlighting the significant risk for their exposed household contacts and healthcare workers [15], although this is not necessarily an indication of viable bacilli. This is also high considering that effective treatment should render MDR-TB patients rapidly non-infectious [16]. Almost a third of routine specimens did not have a smear result documented, an increasingly common scenario in settings where Xpert is used universally for rapid detection of MTB and of rifampicin resistance [17]. Sputum smear microscopy is often done on a second sputum specimen at baseline for treatment monitoring [18]. In the absence of sputum smear results, Xpert cycle threshold values or ranges could be reported to provide a quantitative measure of bacillary load reflecting degree of infectiousness at the time of diagnosis [19]. Such data were not collected for this study.

Our study has several limitations. MDR-TB treatment was provided by the local program and details of patient adherence or gaps in treatment were not available. Additional testing during treatment may also have been performed by the programs, but we only collected results from specimens collected at the time of diagnosis. Finally, some tests could have been done but not recorded as MDR-TB laboratory reports can be complex to interpret, as multiple tests are done and reported over multiple days.

## Conclusion

The mycobacteriology objectives for this study were to determine if MTB was detectable and if resistance to INH and RIF could be confirmed, genotypically and phenotypically, in adult index cases with drug resistant TB at time of enrolment. We found that only three quarters of the index cases had documented MDR-TB and that for the remainder, this could not be confirmed on study for most participants. Despite these limitations, this study provided valuable data and informed improvements to the interventional trial design aimed at evaluating TB preventive therapy to high-risk HHCs. Our data also highlight the challenges and importance of careful and systematic documentation of MDR-TB microbiological results to ensure high-quality data for clinical research and to ensure appropriate TB preventive therapy is offered to close contacts. Finally, our study also has implications for routine care settings in additional to the research context. Capacity building in TB control programs is needed to provide the necessary infrastructure to enable accurate characterization of patients with MDR-TB, to provide them with optimal therapy, and to inform the best strategy prevent TB in their household contacts.

## Data Availability

Data are available to all interested researchers upon request to the Statistical and Data Analysis Center of the AIDS Clinical Trials Group (e-mail: sdac.data@sdac.harvard.edu) and the Statistical and Data Management Center data access committee of the IMPAACT network (email address: sdac.data@fstrf.org) with the written agreement of both networks.

## Abbreviations

ACTG: AIDS Clinical Trials Group
DST: Drug Susceptibility Testing
FLQ: Fluoroquinolones
HHCs: Household Contacts
IMPAACT: International Maternal Pediatric Adolescent Aids Clinical Trials
INH: Isoniazid
IQR: Interquartile Ranges
LPA: Line Probe Assays
MDR-TB: Multidrug-Resistant TB
MTB: *Mycobacterium tuberculosis*
NTM: *Nontuberculous mycobacteria*
Pre-XDR-TB: Pre-Extensively Drug-Resistant TB
RIF: Rifampicin
RR-TB: Rifampicin-Resistant Tuberculosis
SLID: Second-Line Injectable Drugs
XDR-TB: Extensively Drug-Resistant TB
